# Clinical and molecular characterization of a patient with interstitial 6q21q22.1 deletion

**DOI:** 10.1186/s13039-015-0134-7

**Published:** 2015-04-28

**Authors:** Elisa Tassano, Marisol Mirabelli-Badenier, Edvige Veneselli, Aldamaria Puliti, Margherita Lerone, Carlotta Maria Vaccari, Giovanni Morana, Simona Porta, Giorgio Gimelli, Cristina Cuoco

**Affiliations:** Laboratorio di Citogenetica, Istituto Giannina Gaslini, L.goG.Gaslini 5, 16147 Genova, Italy; Child Neuropsychiatry Unit, Department of Neurosciences and Rehabilitation, Istituto Giannina Gaslini, Genoa, Italy; Department of Neurosciences Rehabilitation Ophthalmology Genetics Maternal and Child Health (DiNOGMI), University of Genoa, Genoa, Italy; U.O.C. Genetica Medica, Istituto Giannina Gaslini, Genova, Italy; Pediatric Neuroradiology Unit, Istituto Giannina Gaslini, Genoa, Italy

**Keywords:** Interstitial deletion, 6q21q22.1, Array-CGH, Karyotype/phenotype correlation, Poland syndrome

## Abstract

**Background:**

Interstitial 6q deletions, involving the 6q15q25 chromosomal region, are rare events characterized by variable phenotypes and no clear karyotype/phenotype correlation has been determined yet.

**Results:**

We present a child with a 6q21q22.1 deletion, characterized by array-CGH, associated with developmental delay, intellectual disability, microcephaly, facial dysmorphisms, skeletal, muscle, and brain anomalies.

**Discussion:**

In our patient, the 6q21q22.1 deleted region contains ten genes (*TRAF3IP2, FYN, WISP3, TUBE1, LAMA4, MARCKS, HDAC2, HS3ST5, FRK, COL10A1*) and two desert gene regions. We discuss here if these genes had some role in determining the phenotype of our patient in order to establish a possible karyotype/phenotype correlation.

**Electronic supplementary material:**

The online version of this article (doi:10.1186/s13039-015-0134-7) contains supplementary material, which is available to authorized users.

## Background

Interstitial deletions of the long arm of chromosome 6 are rare and are divided into proximal (6q11q16), medial (6q15q25), and terminal (6q25qter) based on conventional cytogenetics [1]. Approximately, less than 30 patients with intermediate 6q interstitial deletions, studied by standard cytogenetics and array-CGH, have been reported [2-13].

The phenotype of patients with medial 6q deletion is generally associated with intrauterine growth retardation (IUGR), abnormal respiration, hypertelorism, and upper limb malformations [1]. However, the patients described to date presented a large spectrum of clinical features, depending on the size of the deleted segment, the involved genes, and the genomic architecture of the region, making genotype–phenotype correlation difficult.

Here, we report on the phenotypic and molecular characterization of a new 6q21q22.1 deletion in a boy with developmental delay, intellectual disability, microcephaly, facial dysmorphisms, and skeletal, muscle, and brain anomalies. We compare the phenotype of our patient with that of previously reported patients and discuss the role of the deleted genes in order to establish a possible karyotype/phenotype correlation.

## Case presentation

The patient, a 13-year-old boy, is the only child of non-consanguineous, healthy Paraguayan parents. The child was born at term by elective caesarean section after an uneventful pregnancy. Birth weight was 4060 g and no perinatal diseases were reported. Data on the patient’s history as a child are not available because he lived in Paraguay with his grandparents, however delay in psychomotor development was reported (he was able to sit without support at 8 months and to walk at 18 months). At 12 years, he moved to Italy to join his mother and at 13 years he was admitted to our hospital because of mild intellectual disability and dysmorphic features.

**Figure 1:**
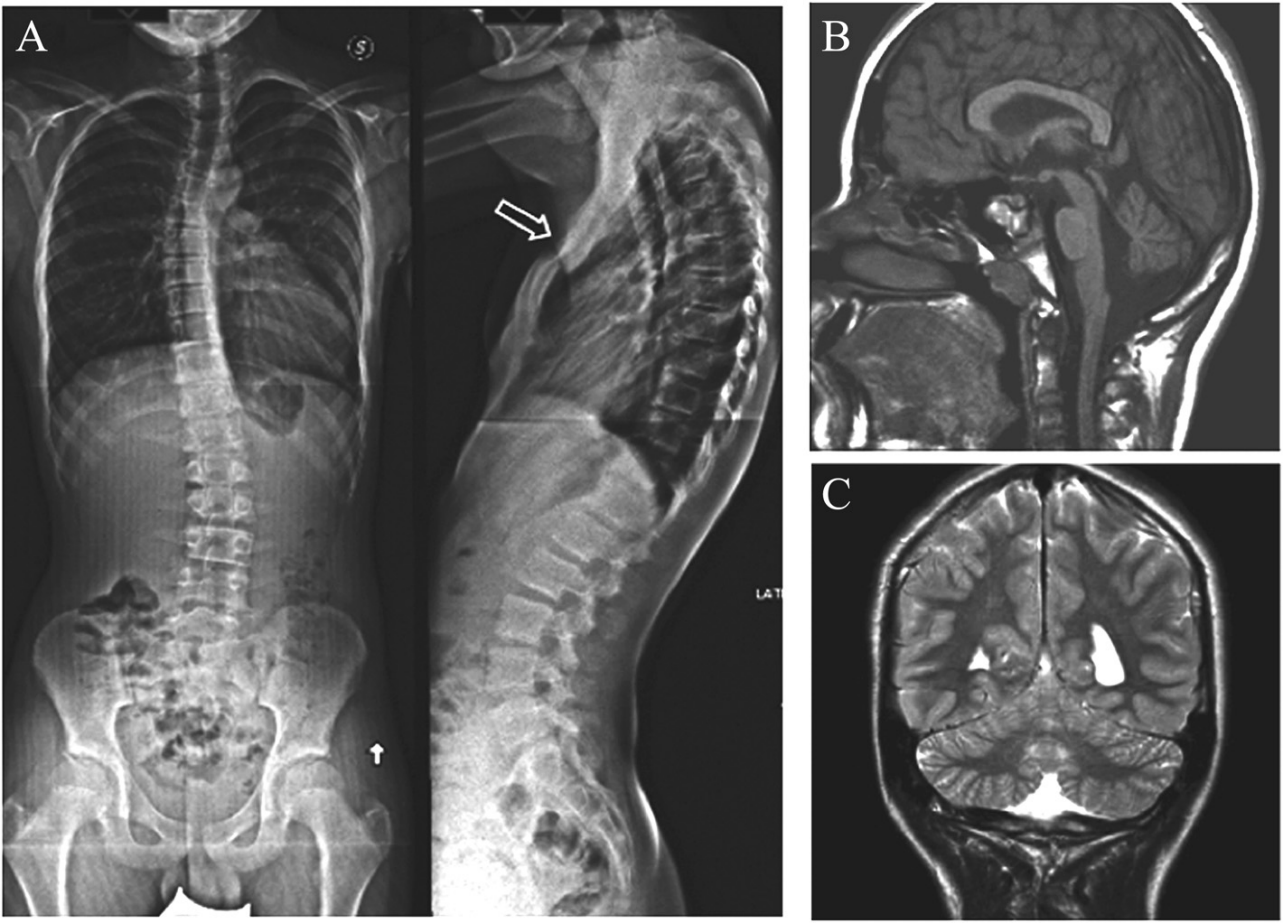
X-ray and MRI analyses of the patient. **A)** Standing X-ray films of full-length spine. Front view showing thoracolumbar scoliosis and side view demonstrating pectus excavatum (open arrow). No associated vertebral abnormalities are present. **B, C**) Brain MRI.) Midsagittal T1-weighted image demonstrating moderate vermis hypoplasia **(B)**. Coronal T2-weighted image showing normal cerebellar hemispheres **(C)**.

Neurological examination demonstrated mild clumsiness without obvious focal signs. On physical examination, his weight was 42.8 Kg (50-75th percentile), height 161 cm (75-90th percentile), and head circumference 52 cm (3rd-10th percentile). He showed facial dysmorphisms (hypertelorism, wide and flat nose), pectus excavatum and chest asymmetry. The absence of pectoralis major and minor muscles on the right side was demonstrated by ultrasound imaging and the diagnosis of Poland Syndrome (PS) (MIM 173800) was made. Standing X-ray films of the full-length spine showed thoracolumbar scoliosis, without vertebral abnormalities (Figure 1A). No additional anomalies of the kidney, urinary tract, or heart were detected by ultrasound examination. Ophthalmologic evaluation demonstrated intermittent exotropia*,* bilateral myopia, and normal fundus oculi. Brain magnetic resonance imaging (MRI) showed cerebellar vermis hypoplasia (Figure 1B, C). Neurophysiological studies including electroencephalogram (EEG) and brainstem auditory evoked potentials (BAEP) resulted normal.

Cognitive impairment was revealed by psychometric evaluation (<5th centile at Raven’s Progressive Matrices P.M.38).

## Results

**Figure 2:**
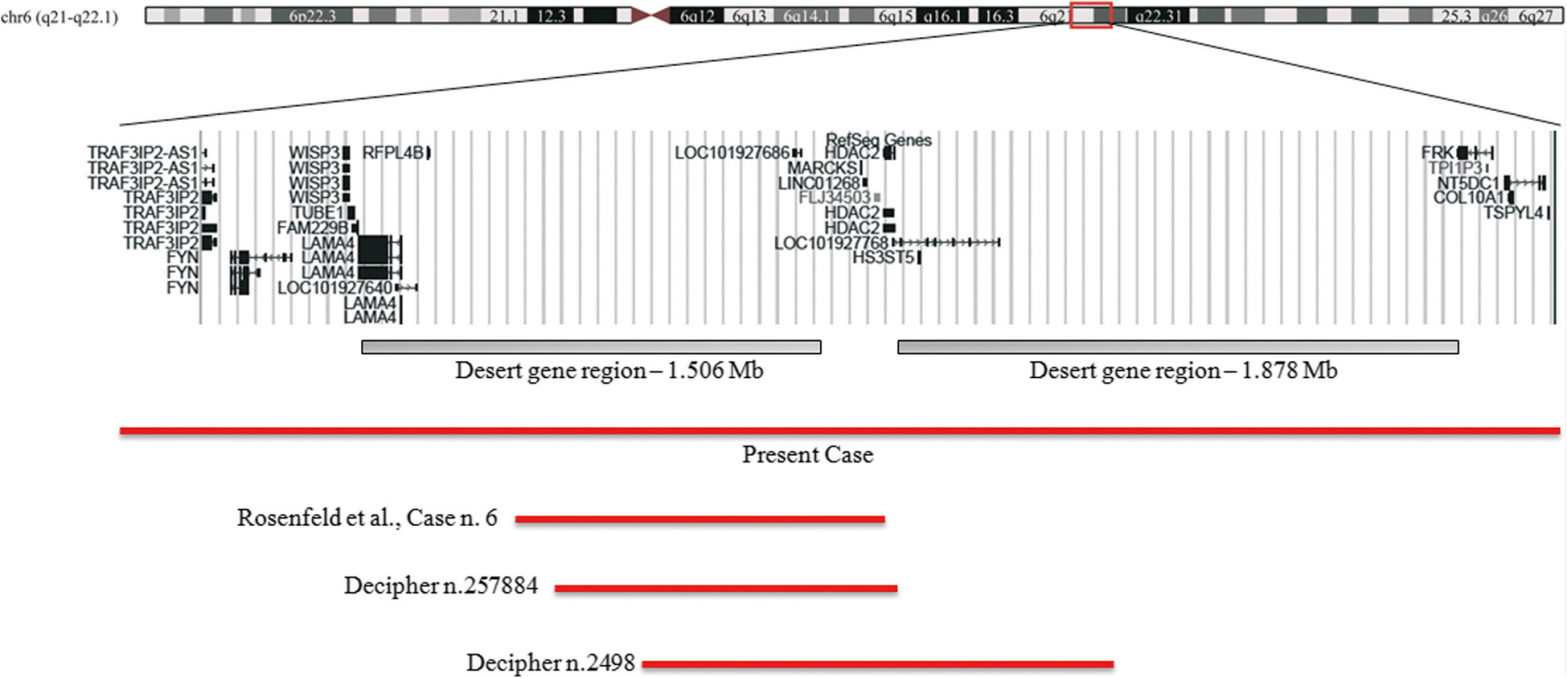
Overview of the region 6q21q22.1 and its gene content according to the UCSC Genome Browser [GRCh37/hg19 assembly]. The bars indicate the deleted region in our patient, in Rosenfeld et al. case n.6, in DECIPHER case n.257884, and in DECIPHER case n.2498.

Cytogenetic analysis, performed on GTG-banded metaphases from cultured lymphocytes of the patient and his mother, showed normal karyotypes. Considering the phenotypic abnormalities of the patient, array-CGH analysis was performed, showing a 4.71 Mb interstitial deletion at 6q21q22.1 bands. The deletion spanned from genomic position 111,884,640 Kb to 116,594,641 Kb (Additional file 1: Figure S1). The 6q21 deleted region includes 8 OMIM genes: *TRAF3IP2* (MIM 607043) traf3-interacting protein 2*; FYN* (MIM 137025) fyn oncogene related to *SRC*, *FGR*, *YES*; *WISP3* (MIM 603400) WNT1-inducible signalling pathway protein 3*;TUBE1* (MIM 607345) tubulin, epsilon-1*; LAMA4* (MIM 600133) laminin, alpha-4*; MARCKS* (MIM 177061*)* myristoylated alanine-rich protein kinase c substrate*; HDAC2* (MIM 605164) histone deacetylase 2*; HS3ST5* (MIM 609407) heparansulfate (glucosamine) 3-o-sulfotransferase 5. The deletion also contains a 1.5 Mb gene desert region that, conversely, is characterized by the presence of several large intergenic non coding RNAs (lincRNAs) and of transcripts of uncertain coding potential (TUCPs), according to The Human Body Map catalogue [14,15]. The 6q22.1 deleted region includes a 1.878 Mb desert region and 2 OMIM genes: *FRK* (MIM 606573) fyn-related kinase; *COL10A1* (MIM 120110) collagen, type X, alpha-1 (Figure 2).

Array-CGH analysis performed on peripheral blood of the mother was normal.

## Discussion

We report on a 13-year-old boy presenting developmental delay, intellectual disability, microcephaly, facial dysmorphisms, and skeletal, muscle, and brain anomalies. Array-CGH identified a 4.71 Mb interstitial deletion at 6q21q22.1 bands. Rosenfeld et al. [8] described 12 individuals with variable deletions within 6q15q22.33 and compared their clinical features to better define karyotype/phenotype correlations. They reported heterogeneous phenotypes, even among individuals with overlapping deletions. They speculated that phenotypic variability could be related to less penetrance, concomitant mutations in other genes, in non-coding regions, such as transcription factor binding sites, or in methylation patterns.

Searching for patients with similar chromosomal imbalances, we select the patient n.6 reported by Rosenfeld et al. (2012) [8] and, in the DECIPHER database (DECIPHER, https://decipher.sanger.ac.uk/), another 2 patients (n.257884, n.2498) sharing part of the deleted region with ours.

A comparison of our patient’s phenotype with those reported in the literature and in DECIPHER database is shown in Table 1. All the patients were studied by array-CGH. Microcephaly, developmental delay, intellectual disability, and skeletal and ophthalmologic features were common among these patients. Interestingly, our patient also showed some features of Poland syndrome (PS) as the absence of pectoralis major and minor muscles on the right side.

**Table 1:** **Phenotype and molecular comparison**

	**Our case**	**Case n.6 Rosenfeld et al. 2012**	**Decipher n. 257884**	**Decipher n. 2498**
Gender	M	M	F	ND
Age	12 years	12 years	5 years	ND
Deletions (UCSC hg.19)	chr6:111,884,640-116,594,641	chr6:113,083,368-114,343,715	chr6:113,044,405-114,771,988	chr6:113,106,907-115,325,935
Inheritance	Mother normal/father not available	Unknown	*De novo*	ND
Weight	50-75th	55th	−2 DS (14 kg)	+0.4 SD
Height	75-90th	50th	−1 DS (104 cm)	−1.2 SD
OFC	3rd-10th	−1,4 SD	−2 DS (48 cm)	−7.2 SD
DD	+	+	+	+
ID	+	+	+	+
Hypotonia	-	-	Moderate	Right hemiplegia with brisk reflexes and spasticity
Brain Malformation	Cerebellar vermis hypoplasia	-	Corpus callosum hypoplasia	Reduction in volume in the left hemi cranium with cystic cerebromalacia.
			Cortical dysplasia	Thin corpus callosum
Other neurological features	-	-	Dysexecutive syndrome	Epilepsy
Ophtalmologic features	Exotropia, bilateral myopia	Strabismus	Strabismus (exophoria/exotropia)	Moderate cerebral visual impairment
Dysmorphic features	Hypertelorism, wide and flat nose	Hypertelorism, wide nasal bridge, narrow nasal tip, long nose	Triangular face, retrognathism, low set ears, smooth philtrum	Hypertelorism, prominent simple ears
Limbs	-	Long and slender fingers and toes	ND	-
Skeletal features	Pectus excavatum, chest asymmetry, thoracic scoliosis and vertebral rotation	Pectus carenatum	ND	Required reconstruction of right hip probably secondary to hemiplegia
Muscle features	Absence of the pectoralis major and minor muscles (Poland Syndrome)	-	ND	-
Other	-	Hyperextensible joints		Constipation
Deleted region	chr6:111,884,640-116,594,641	chr6:113,190,061-114,450,408	chr6:113,044,405-114,771,988	chr6:113,106,907-115,325,935
Genes	*TRAF3IP2, FYN, WISP3, TUBE1, LAMA4, MARCKS, HDAC2, HS3ST5, FRK, COL10A1*	*MARCKS, HDAC2, HS3ST5*	*MARCKS, HDAC2, HS3ST5*	*MARCKS, HDAC2, HS3ST5*

ND = not determined; OFC = Occipitofrontal Circumference; DD = developmental delay; ID = intellectual disability.

We consider the deleted region (chr6:113,190,061-114,450,408) shared by our case, patient n.6 [8], and the two DECIPHER cases, n.257884 and n.2498 (Figure 2). It contains three genes: *MARCKS, HDAC2,* and *HS3ST5.* It is known that *MARCKS* (myristoylated alanine-rich C kinase substrate) encodes an actin cross-linking protein that plays a role in signal transduction pathways, postnatal survival, cellular migration and adhesion, as well as endo-, exo-, and phagocytosis, and neurosecretion. Moreover, MARCKS is expressed in brain and spinal cord from the early stages of development. It is required during embryogenesis, as revealed by several gene knock-out studies: mice heterozygous for MARCKS appear normal, but exhibit impaired spatial learning while mice lacking the entire MARCKS gene show severe abnormalities of the central nervous system, and all die around birth [16].

The *HDAC2* (Histone deacetylase 2) gene encodes a transcription factor that enhances cognitive ability, corrects neurodegenerative impairment, and helps to re-establish long-term memory [17].

*HS3ST5* gene encodes a protein that belongs to a group of heparansulfate 3-O-sulfotransferases highly expressed in fetal brain, followed by adult brain and spinal cord [18].

Since these three genes are involved in neural development, we could speculate that their deletion could cause neurological phenotypes like developmental delay, intellectual disability, and brain malformations, all observed in our patient and in the other three similar patients as shown in Table 1.

Moreover, in our patient, the genomic 6q21q22.1 deleted region contained another seven OMIM genes, including *FYN, WISP3,*and *COL10A1. FYN* is a non-receptor tyrosine kinase belonging to the Src family kinases. It proved to play important roles in neuronal functions, including myelination and oligodendrocyte formation, and in inflammatory processes [19]. *WISP3* encodes a member of the CCN (connective tissue growth factor, Cysteine-rich 61, nephroblastoma overexpressed) family of connective tissue growth factor, known to be mostly extracellular matrix-associated proteins, involved in regulation of cell migration and adhesion, cell proliferation, differentiation, and survival in connective tissues. It is expressed in skeletal-derived cells, such as synoviocytes, chondrocytes, and bone marrow-derived mesenchymal progenitor cells, and it is involved in skeletal development and maintenance of cartilage integrity [20]. *COL10A1* encodes type X collagen specifically expressed by hypertrophic chondrocytes. As a major component of the hypertrophic zone, type X collagen influences the deposition of other matrix molecules in this region, thereby providing a proper environment for haematopoiesis, mineralization, and modelling, that are essential for endochondral ossification. Mutations and abnormal expression of *COL10A1* are closely linked to abnormal chondrocyte hypertrophy, which has been observed in multiple skeletal dysplasia and osteoarthritis [21]. For these reasons, we could speculate that, in our patient, haploinsufficiency of *FYN* could have contributed to neurological anomalies, and *WISP3* and *COL10A1* to skeletal defects.

To date, the pathogenic mechanisms underlying PS are still unknown and the genetic origin of the disease is still a matter of debate. It has been hypothesized that PS defects could result from a vascular insult during early embryological stages, which implies that environmental factors could contribute to PS phenotype [22,23].

## Conclusions

Interstitial 6q deletion can cause a variable phenotype depending on the size and location of the anomaly. Our paper may contribute to a better understanding of karyotype/phenotype correlation in cases with deletion in 6q21q22.1 and to determine the clinical implication of the genes present in the involved chromosomal region. Identification of additional individuals with overlapping interstitial deletion will help to better define this correlation.

## Methods

Standard GTG banding was performed at a resolution of 400–550 bands on metaphase chromosomes from peripheral blood lymphocytes of the patient and his mother; the father refused any analysis. Molecular karyotyping was performed in the patient and his mother using Human Genome CGH Microarray Kit G3 180 (Agilent Technologies, Palo Alto, USA) with ~13 Kb overall median probe spacing. Labelling and hybridization were performed following the protocols provided by the manufacturers. A graphical overview was obtained using Agilent Genomic Workbench Lite Edition Software 6.5.0.18.

## Consent

Written informed consent was obtained from the patient’s parents for publication of this paper and any accompanying images. A copy of the written consent is available for review by the Editor-in-Chief of this journal.

## Additional file

Additional file 1: Figure S1. Array-CGH profile. Array-CGH shows a ~4.71 Mb interstitial deletion at 6q21q22.1 bands. The deletion spanned from genomic position 111,884,640 Kb to 116,594,641 Kb. The deleted region contains 11 OMIM genes and two gene desert regions of ~1.5 Mb and ~1.8 Mb, respectively.
