# Supplementary figures and images for: Clinical and molecular characterization of a patient with interstitial 6q21q22.1 deletion
Source: Mol Cytogenet. 2015 Apr 28;8:31. doi: 10.1186/s13039-015-0134-7 (PMC4457201; doi:10.1186/s13039-015-0134-7)

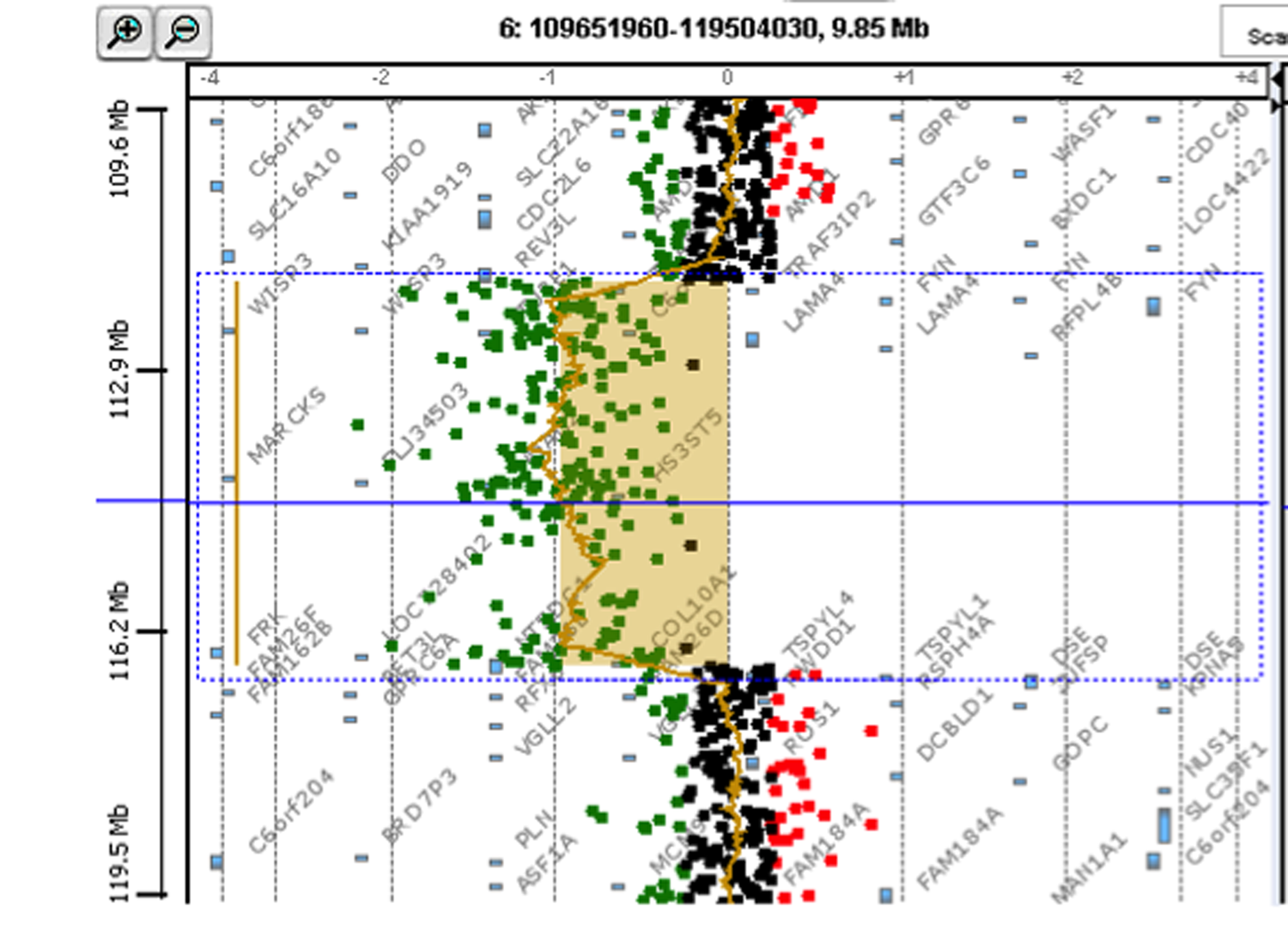

Supplement: Additional file 1: Figure S1. — Array-CGH profile. Array-CGH shows a ~4.71 Mb interstitial deletion at 6q21q22.1 bands. The deletion spanned from genomic position 111,884,640 Kb to 116,594,641 Kb. The deleted region contains 11 OMIM genes and two gene desert regions of ~1.5 Mb and ~1.8 Mb, respectively. [file 13039_2015_134_MOESM1_ESM.tiff]
